# Persisting Sex Discrepancies in Short-Term Outcomes of Patients with ST-Segment Myocardial Infarction: Results of the ISACS-STEMI COVID-19 Registry

**DOI:** 10.3390/jcm15103560

**Published:** 2026-05-07

**Authors:** Giuseppe De Luca, Stephane Manzo-Silberman, Filippo Zilio, Magdy Algowhary, Berat Uguz, Dinaldo C. Oliveira, Vladimir Ganyukov, Zan Zimbakov, Miha Cercek, Lisette Okkels Jensen, Poay Huan Loh, Lucian Calmac, Gerard Roura i Ferrer, Alexandre Quadros, Marek Milewski, Fortunato Scotto D’Uccio, Clemens von Birgelen, Francesco Versaci, Jurrien Ten Berg, Gianni Casella, Aaron Wong Sung Lung, Petr Kala, José Luis Díez Gil, Xavier Carrillo, Maurits Dirksen, Victor M. Becerra-Munoz, Michael Kang-yin Lee, Dafsah Arifa Juzar, Rodrigo de Moura Joaquim, Roberto Paladino, Davor Milicic, Periklis Davlouros, Nikola Bakraceski, Luca Donazzan, Adriaan Kraaijeveld, Gennaro Galasso, Lux Arpad, Lucia Marinucci, Vincenzo Guiducci, Maurizio Menichelli, Alessandra Scoccia, Aylin Hatice Yamac, Kadir Ugur Mert, Xacobe Flores Rios, Tomas Kovarnik, Michal Kidawa, Josè Moreu, Vincent Flavien, Enrico Fabris, Iñigo Lozano Martínez-Luengas, Francisco Bosa Ojeda, Robert Rodríguez-Sanchez, Gianluca Caiazzo, Giuseppe Cirrincione, Hsien-Li Kao, Juan Sanchis Forés, Luigi Vignali, Helder Pereira, Santiago Ordoñez, Alev Arat Özkan, Bruno Scheller, Heidi Lehtola, Rui Teles, Christos Mantis, Ylitalo Antti, João António Brum Silveira, Rodrigo Zoni, Ivan Bessonov, Stefano Savonitto, George Kochiadakis, Dimitrios Alexopulos, Carlos E. Uribe, John Kanakakis, Benjamin Faurie, Gabriele Gabrielli, Alejandro Gutierrez Barrios, Juan Pablo Bachini, Alex Rocha, Frankie Chor-Cheung Tam, Alfredo Rodriguez, Antonia Anna Lukito, Anne Bellemain-Appaix, Gustavo Pessah, Giuliana Cortese, Guido Parodi, Mohammed Abed Burgadha, Elvin Kedhi, Pablo Lamelas, Harry Suryapranata, Matteo Nardin, Monica Verdoia

**Affiliations:** 1 Division of Cardiology, AOU “Policlinico G. Martino”, Department of Clinical and Experimental Medicine, University of Messina, 98121 Messina, Italy; 2 Division of Cardiology, IRCSS Galeazzi-Sant’Ambrogio Hospital, 20157 Milan, Italy; 3 Department of Cardiology, Pitié-Salpêtrière Hospital, Institute of Cardiology, ACTION Study Group, Sorbonne University, 75005 Paris, France; stephane.manzosilberman@aphp.fr; 4 Division of Cardiology, Ospedale Santa Chiara, 38122 Trento, Italy; filippozi@yahoo.it; 5 Division of Cardiology, Assiut University Heart Hospital, Assiut University, Assiut 71524, Egypt; magdyalgowhary@aun.edu.eg; 6 Division of Cardiology, Bursa City Hospital, 16000 Bursa, Turkey; beratuguz33@hotmail.com; 7 Pronto de Socorro Cardiologico Prof. Luis Tavares, Centro PROCAPE, Federal University of Pernambuco, Recife 50670-901, Brazil; dinaldo@cardiol.br; 8 Department of Heart and Vascular Surgery, State Research Institute for Complex Issues of Cardiovascular Diseases, 650000 Kemerovo, Russia; ganyukov@mail.ru; 9 University Clinic for Cardiology, Medical Faculty, Ss’ Cyril and Methodius University, 1000 Skopje, North Macedonia; i.mitevskapeovskai@yahoo.com; 10 Centre for Intensive Internal Medicine, University Medical Centre, 1000 Ljubljana, Slovenia; miha.cercek@gmail.com; 11 Division of Cardiology, Odense Universitets Hospital, 5230 Odense, Denmark; lisette.okkels.jensen@rsyd.dk; 12 Department of Cardiology, National University Hospital, Singapore 119074, Singapore; poay_huan_loh@nuhs.edu.sg; 13 Clinic Emergency Hospital, 050098 Bucharest, Romania; lcalmac@gmail.com; 14 Interventional Cardiology Unit, Heart Disease Institute, Hospital Universitari de Bellvitge, 08907 Barcelona, Spain; groura@bellvitgehospital.cat; 15 Instituto de Cardiologia do Rio Grande do Sul, Porto Alegre 90620-001, Brazil; a.quadros@otahos.ar; 16 Division of Cardiology, Medical University of Silezia, 40-002 Katowice, Poland; marek.milewski92@gmail.com; 17 Division of Cardiology, Ospedale del Mare, 80147 Napoli, Italy; scottof@libero.it; 18 Department of Cardiology, Medisch Spectrum Twente, Thoraxcentrum Twente, 7512 KZ Enschede, The Netherlands; c.vonbirgelen@mst.nl; 19 Division of Cardiology, Ospedale Santa Maria Goretti, 04140 Latina, Italy; francescoversaci@yahoo.it; 20 Division of Cardiology, St Antonius Hospital, 3435 CM Nieuwegein, The Netherlands; j.ten.berg@antoniusziekenhuis.nl; 21 Division of Cardiology, Ospedale Maggiore, 40100 Bologna, Italy; gianni.casella@ausl.bologna.it; 22 Department of Cardiology, National Heart Center, Singapore 169609, Singapore; aaron.wong.s.l@singhealth.com.sg; 23 University Hospital Brno, Medical Faculty of Masaryk University, 63500 Brno, Czech Republic; kala.petr@fnbrno.cz; 24 Department of Cardiology, H. Universitario y Politécnico La Fe, 46026 Valencia, Spain; diez_jlu@gva.es; 25 Department of Cardiology, Hospital Germans Triasi Pujol, 08916 Badalona, Spain; xcarrillosuarez@gmail.com; 26 Division of Cardiology, Northwest Clinics, 1815 JD Alkmaar, The Netherlands; m.t.dirksen@nwz.nl; 27 Department of Cardiology, Hospital Clínico Universitario Virgen de la Victoria, 291010 Málaga, Spain; vmbecerram@gmail.com; 28 Department of Cardiology, Queen Elizabeth Hospital, University of Hong Kong, Hong Kong 999077, China; leekym@ha.org.hk; 29 Department of Cardiology and Vascular Medicine, University of Indonesia National Cardiovascular Center “Harapan Kita”, Jakarta 11420, Indonesia; djuzar@gmail.com; 30 Department of Cardiology, Instituto de Cardiologia de Santa Catarina Praia Comprida, São José 88103-700, Brazil; rodrigojoaquim@gmail.com; 31 Division of Cardiology, Clinica Villa dei Fiori, 80011 Acerra, Italy; brauss@libero.it; 32 Department of Cardiology, University Hospital Centre, University of Zagreb, 10000 Zagreb, Croatia; d.milicic@mail.inet.hr; 33 Invasive Cardiology and Congenital Heart Disease, Patras University Hospital, 26504 Patras, Greece; pdav@med.upatras.gr; 34 Division of Cardiology Center for Cardiovascular Diseases, 6000 Ohrid, North Macedonia; nbakrac@yahoo.com; 35 Division of Cardiology, Ospedale “S. Maurizio”, 39100 Bolzano, Italy; luca.donazzan@sabes.it; 36 Division of Cardiology, University Medical Center, 3684 CX Utrecht, The Netherlands; a.o.kraaijeveld-3@umcutrecht.nl; 37 Division of Cardiology, Ospedale San Giovanni di Dio e Ruggi d’Aragona, 84131 Salerno, Italy; ggalasso@unisa.it; 38 Division of Cardiology, Maastricht University Medical Center, 6229 HX Maastricht, The Netherlands; arpad.lux@mumc.nl; 39 Division of Cardiology, Azienda Ospedaliera “Ospedali Riuniti Marche Nord”, 61121 Pesaro, Italy; lucia.marinucci@alice.it; 40 Division of Cardiology, AUSL-IRCCS, 42122 Reggio Emilia, Italy; vincenzo.guiducci@ausl.re.it; 41 Division of Cardiology, Ospedale “F. Spaziani”, 03100 Frosinone, Italy; menichelli747@gmail.com; 42 Division of Cardiology, Ospedale “Sant’Anna”, 44124 Ferrara, Italy; scoccia.alessandra@gmail.com; 43 Department of Cardiology, Hospital Bezmialem Vakıf University, 34093 Istanbul, Turkey; aylin-yamac@yandex.com; 44 Division of Cardiology, Faculty of Medicine, Eskisehir Osmangazi University, 26040 Eskisehir, Turkey; kugurmert@gmail.com; 45 Department of Cardiology, Complexo Hospetaliero Universitario La Coruna, 15006 La Coruna, Spain; acobe.flores.rios@sergas.es; 46 Department of Cardiology, University Hospital, 12808 Prague, Czech Republic; tomas.kovarnik@vfn.cz; 47 Department of Cardiology, Central Hospital of Medical University, 91-425 Lodz, Poland; kidawa@ptkardio.pl; 48 Division of Cardiology, Complejo Hospitalario de Toledo, 45005 Toledo, Spain; jmoreu@sescam.jccm.es; 49 Division of Cardiology, Center Hospitalier Universitaire de Lille, 59000 Lille, France; flavienvincent@yahoo.fr; 50 Azienda Ospedaliero—Universitaria Ospedali Riuniti, 34142 Trieste, Italy; enrico.fabris@hotmail.it; 51 Division of Cardiology, Hospital Cabueñes, 33394 Gijon, Spain; inigo.lozano@gmail.com; 52 Division of cardiology, Hospital Universitario de Canarias, 38320 Santa Cruz de Tenerife, Spain; franbosa@ull.edu.es; 53 Division of Cardiology, Hospital Puerta de Hierro, 28222 Majadahonda, Spain; arellanoserrano@serhosp.es; 54 Division of Cardiology, Ospedale “G Moscati”, 81031 Aversa, Italy; gianluca.caiazzo@gmail.com; 55 Division of Cardiology, Ospedale Civico Arnas, 90127 Palermo, Italy; giuseppe_cirri@arnas.it; 56 Cardiology Division, Department of Internal Medicine, National Taiwan University Hospital, Tapei 100229, Taiwan; hsienli_kao@yahoo.com; 57 Division of Cardiology, Hospital Clinico Universitario, 46010 Valencia, Spain; sanchis_juafor@gva.es; 58 Interventional Cardiology Unit, Azienda Ospedaliera Sanitaria, 43126 Parma, Italy; luvignali@ao.pr.it; 59 Cardiology Department, Hospital Garcia de Orta (Pragal), 2805-267 Almada, Portugal; hhpereira@gmail.com; 60 Instituto Cardiovascular de Buenos Aires, Buenos Aires 1428, Argentina; sordonez@icba.com.ar (S.O.); plamelas@icba.com.ar (P.L.); 61 Cardiology Institute, Instanbul University, 34093 Instanbul, Turkey; alevarat@hotmail.com; 62 Division of Cardiology, Clinical and Experimental Interventional Cardiology, University of Saarland, 66123 Saarbrucken, Germany; bruno.scheller@uks.eu; 63 Division of Cardiology, Oulu University Hospital, 90220 Oulu, Finland; heidi.lehtola@ppshp.fi; 64 Division of Cardiology, Hospital de Santa Cruz, CHLO—Nova Medical School, Centro de Estudos de Doenças Crónicas (CEDOC), 2790-134 Lisbon, Portugal; rcteles@outlook.com; 65 Division of Cardiology, Kontantopoulion Hospital, 142 33 Athens, Greece; vardas.e@heraklin.gr; 66 Division of Cardiology, Heart Centre Turku, P.O. Box 52, 20521 Turku, Finland; antti.ylitalo@tyks.fi; 67 Division of Cardiology, Hospital de Santo António, 4050-522 Porto, Portugal; brumsilveira@gmail.com; 68 Department of Teaching and Research, Instituto de Cardiología de Corrientes “Juana F. Cabral”, Corrientes 3400, Argentina; rodrizoni@yahoo.com.ar; 69 Tyumen Cardiology Research Center, 625026 Tyumen, Russia; bessonov@infarkta.net; 70 Division of Cardiology, Ospedale “A. Manzoni”, 23900 Lecco, Italy; s.savonitto@ospedale.lecco.it; 71 Iraklion University Hospital, 711 10 Crete, Greece; j.kochiadachis@gmail.com; 72 Division of Cardiology, Attikon University Hospital, 124 62 Athens, Greece; dalex@med.uoa.gr; 73 Division of Cardiology, CES, Universidad UPB, 050031 Medellin, Colombia; uribemd72@hotmail.com; 74 Division of Cardiology, Alexandra Hospital, 115 28 Athens, Greece; jkanakakis@yahoo.gr; 75 Division of Cardiology, Groupe Hospitalier Mutualistee, 38000 Grenoble, France; faurieb@gmail.com; 76 Interventional Cardiolgy Unit IRCCS INRCA, 030034 Ancona, Italy; g_gabrielli@yahoo.it; 77 Division of Cardiology, Hospital Puerta del Mar, 11009 Cadiz, Spain; 78 Instituto de Cardiologia Integral, Montevideo 11700, Uruguay; drbachini@gmail.com; 79 Department of Cardiology and Cardiovascular Interventions, Instituto Nacional de Cirugía Cardíaca, Montevideo 11700, Uruguay; alexrocha@hemodinamia.onmicrosoft.com; 80 Department of Cardiology, Queen Mary Hospital, University of Hong Kong, Hong Kong 999077, China; frankie.tamcc@gmail.com; 81 Division of Cardiology, Otamendi Hospital, Buenos Aires 1001, Argentina; arodriguez@centroceci.com.ar; 82 Cardiovascular Department, Siloam Lippo Village Hospital, Heart Center, Pelita Harapan University, Tangerang 15810, Indonesia; antonia.lukito@uph.edu; 83 Cardiology, Center Hospitalier d’Antibes Juan Les Pins, 06600 Antibes, France; veauthyelau@jlespins.fr; 84 Division of Cardiology, Hospital Cordoba, Cordoba 5000, Argentina; pessah.g@cordoba.es; 85 Department of Statistical Sciences, University of Padova, 35122 Padova, Italy; gcortese@stat.unipd.it; 86 Department of Cardiology, Azienda Ospedaliero-Universitaria, 001579 Sassari, Italy; parodiguido@gmail.com; 87 Division of Cardiology, Blida University Hospital, Blida 09000, Algeria; bouraghda5@yahoo.fr; 88 Department of Interventional Cardiology, Royal Victoria Hospital, McGill University Health Centre, McGill University, Montreal, QC H3T 1J4, Canada; ekedhi@me.com; 89 Division of Cardiology, Radboud University Medical Center, 6525 GA Nijmegen, The Netherlands; suryapranatamd@gmail.com; 90 Department of Internal Medicine, Ospedale Riuniti, 25123 Brescia, Italy; mecionardino@hotmail.com; 91 Division of Cardiology, Ospedale Degli Infermi, ASL Biella, 13900 Biella, Italy; monica.verdoia@gmail.com

**Keywords:** sex, ST-segment elevation myocardial infarction, primary percutaneous coronary intervention, outcomes

## Abstract

**Background**. Despite technological innovations and improvements in stents and devices, sex-related discrepancies are still reported in the outcomes after ST-segment elevation myocardial infarction (STEMI), depending on biological and sex-specific pathophysiological differences, which have not been completely understood. The aim of the present study was to provide real-world data on the prognostic role of sex among patients with STEMI, enclosed into a recent up-to-date international registry. **Methods**. The ISACS-STEMI COVID-19 is a large-scale retrospective registry, including STEMI patients treated with mechanical reperfusion between 1 March and 30 June, 2019 and 2020. Patients, treated in 109 centers across Europe, Latin America, Southeast Asia, and North Africa, were grouped according to sex. Primary endpoint: In-hospital mortality; secondary endpoints: Time delay, 30-day mortality, and postprocedural Thrombolysis In Myocardial Infarction (TIMI) 3 flow. **Results**. We included 16,083 patients, 24.3% females (54.3% hospitalized in 2019, 45.7% in 2020). Women with STEMI were older, more often diabetic and hypertensive (*p* < 0.001), with a higher prevalence of hypercholesterolemia (*p* = 0.02), longer ischemia time (*p* = 0.01), ambulance referral (*p* = 0.03) and cardiogenic shock at presentation (*p* = 0.05), but less frequently smokers, with a previous cardiovascular event (*p* < 0.001) or anterior STEMI (*p* = 0.03) as compared to males. Preprocedural TIMI 0 flow, multivessel disease, need for thrombectomy (*p* < 0.001 and *p* = 0.001, respectively), use of Glycoprotein IIbIIIa inhibitors or cangrelor, radial access and implantation of drug-eluting stents (*p* < 0.001, *p* < 0.001 and *p* = 0.001, respectively) were also more common in men. Impaired postprocedural epicardial reperfusion (TIMI flow 0–2) was observed more frequently in females as compared to males (10% vs. 7.2%; adjusted OR [95% CI] = 1.30 [1.13–1.49], *p* = 0.01). In-hospital mortality was 5.8%, significantly higher among women (8.3% vs. 5%, *p* < 0.001, adjusted HR [95% CI] = 1.26 [1.06–1.5], *p* = 0.01). Similar data were observed for 30-day mortality (10.3% vs. 6.2%, *p* < 0.001, adjusted HR [95% CI] = 1.22 [1.06–1.38], *p* = 0.007). **Conclusions**. Among STEMI patients being treated with the most updated standard of care for primary percutaneous coronary intervention, female sex is still associated with higher complexity and impaired prognosis, displaying suboptimal epicardial reperfusion and increased in-hospital and 30-day mortality.

## 1. Background

Technological innovations and improvements in percutaneous coronary interventions (PCI), with low-thrombogenicity drug-eluting stents (DESs) or even no-stent techniques [1,2,3,4], along with the spread of circulatory support devices [5], have completely revolutionized the outcomes of patients with ST-segment elevation myocardial infarction (STEMI). Nevertheless, the same reduction in mortality has not been observed in females as compared to male patients.

Despite data from large registries and randomized clinical trials consistently reporting sex-related prognostic discrepancies, most of the studies have pointed at the role of other factors, such as comorbidities, delayed presentation and bleeding complications [6,7], rather than sex per se, in conditioning the outcomes after STEMI. In fact, a great level of interest has been focused in recent years on sex-related differences in the pathophysiology of atherosclerosis and in the response to antithrombotic therapies [8,9,10,11,12,13,14,15].

The initiatives and campaigns performed to increase the awareness of patients and clinicians about the cardiovascular risk in women [16,17,18,19] have partially narrowed this gap. In fact, recent large-scale registries with newer DES, which allow for a shorter duration of dual antiplatelet therapy and a lower risk of lesion failure, have shown no sex-related differences in clinical outcomes between men and women [20,21]. Therefore, different results could be expected on the impact of female sex in STEMI patients undergoing primary PCI with the best contemporary standard of care.

The International Study on Acute Coronary Syndromes—ST Elevation Myocardial Infarction (ISACS-STEMI) COVID-19 is a global registry, conducted in 109 high-volume tertiary centers on 4 continents [22,23,24]. It was established in response to the emerging outbreak of COVID-19 and estimated the true impact of the COVID-19 pandemic on the treatment and clinical outcomes of STEMI patients who underwent primary angioplasty.

In the present analysis, we aim to provide an insight into the prognostic impact of sex on the outcomes of patients with STEMI undergoing primary PCI, enclosed in a real-world and up-to-date registry that spans the period of the COVID-19 pandemic.

## 2. Methods

### 2.1. Study Design and Population

The International Study on Acute Coronary Syndromes—ST Elevation Myocardial Infarction (ISACS-STEMI) COVID-19 is a large-scale multicenter retrospective registry, promoted by the Eastern Piedmont University in Novara, Italy, which included STEMI patients treated with primary PCI in Europe (phase 1; [22,23]) and Latin America, Southeast Asia, and North Africa (phase 2; [24]) for a total of in 109 high-volume centers. The final inclusion period was from 1 March 2020 to 30 June 2020. The data were compared with those retrospectively collected during the same months in 2019. Inclusion and exclusion criteria have been previously reported [22]. In brief, we included consecutive STEMI patients treated by primary PCI (including mechanical reperfusion for failed thrombolysis) within the period of study; patients not undergoing invasive treatment or with incomplete in-hospital data were excluded.

*Data Collection.* Anonymized data were collected through a dedicated Case Report Form (CRF). Each center identified a local Principal Investigator. We collected demographic, clinical, and procedural data, including total ischemia and door-to-balloon time (from arrival to hub to balloon inflation), referral to primary PCI facility, COVID-19 positivity, PCI procedural data, and in-hospital mortality. After data collection, each participating center submitted the CRF to the coordinating unit at Eastern Piedmont University, which was responsible for compiling all the data into the central electronic database. Data were checked for missing or contradictory entries.

### 2.2. Study Endpoints

The primary study outcome was in-hospital mortality. Secondary study outcomes were patient-related time delay, postprocedural Thrombolysis In Myocardial Infarction (TIMI) flow < 3 and 30-day mortality.

**Statistics.** Data were analyzed using SPSS Statistics Software 23.0 (IBM SPSS Inc., Chicago, IL, USA) and R software (version 3.6.2) by an independent statistician (GC). Continuous variables were described using median and interquartile range, whereas categorical ones were considered as percentages. Patients were grouped according to sex.

Analysis of variance (ANOVA) and the chi-square test were used for continuous and categorical variables, respectively. Normal distribution of continuous variables was tested by the Kolmogorov–Smirnov test. In the case of non-normally distributed variables, the Mann–Whitney test was applied.

Multivariate logistic regression was performed to evaluate the association between sex and secondary endpoints after correction for baseline confounders (age, diabetes, hypertension, hypercholesterolemia, smoke, previous cardiovascular event, time to reperfusion, ambulance referral, cardiogenic shock, STEMI location, preprocedural TIMI flow, multivessel disease, use of thrombectomy, use of GpIIbIIIa or cangrelor, radial access, and DES), which were entered in the model “in block” (*p*-value entry < 0.05; *p*-value removal > 0.1). Cox regression and Kaplan–Meier estimates were applied to evaluate the association between sex and mortality, after correction for baseline differences (all variables with *p* < 0.05 at univariate analysis, as in Table 1 and Table 2).

A further outcome analysis was conducted to explore the impact of sex on outcomes across age decades (from 55 to 85 years) and to assess the age–sex interaction.

**Ethical issues.** The study was approved by the Ethical Committee in Novara, Italy, and followed the World Medical Association’s Declaration of Helsinki. Due to the retrospective study design, no informed consent was required, as approved by the Ethical Committee and relevant local ethical authorities (Trial registration number: NCT 04412655; CE 132/2020).

## 3. Results

The final population was represented by 16,083 patients [24] (96.4% with complete clinical data out of 16,674 patients), of whom 3919 (24.3%) were females. Among them, 2127 (54.3%) were from 2019, while 1792 (45.7%) were hospitalized in 2020, not resulting in any significant difference (*p* = 0.78).

As shown in Table 1, women with STEMI were older and more often diabetic and hypertensive (*p* < 0.001), with a higher prevalence of hypercholesterolemia (*p* = 0.02), but less frequently smokers or admitted with a previous cardiovascular event (*p* < 0.001) as compared to males.

**Table 1:** Baseline demographic and clinical characteristics.

	Total Population (*n* = 16,083)	Males (*n* = 12,164)	Females (*n* = 3919)	*p*-Value
Age (median, IQR)	63 [54–72]	61 [53–70]	68 [59–78]	<0.001
Elderly (>75 y)—*n* (%)	3047 (18.9)	1772 (14.6)	1275 (32.5)	<0.001
Hypertension—*n* (%)	8813 (54.8)	6271 (51.6)	2542 (64.9)	<0.001
Diabetes mellitus—*n* (%)	3812 (23.7)	2686 (22.1)	1126 (28.7)	<0.001
Hypercholesterolemia—*n* (%)	6353 (39.5)	4741 (39)	1612 (41.1)	0.02
Smokers—*n* (%)	8918 (55.4)	7311 (60.1)	1607 (41)	<0.001
Family history of CAD—*n* (%)	3298 (20.5)	2521 (20.7)	777 (19.8)	0.23
Previous STEMI—*n* (%)	1543 (9.6)	1255 (10.3)	288 (7.3)	<0.001
Previous PCI—*n* (%)	1993 (12.4)	1636 (13.4)	357 (9.1)	<0.001
Previous CABG—*n* (%)	272 (1.7)	218 (1.8)	54 (1.4)	0.09
***Hospital Access***				
Ambulance referral—*n* (%)	7738 (48.1)	5791 (47.6)	1947 (49.7)	0.03
***Time Delays***				
Ischemia time, median [25–75th]	210 [122–378]	190 [120–340]	240 [140–433]	
Total ischemia time				<0.001
<6 h—*n* (%)	11,922 (74.1)	9141 (75.1)	2781 (71)	
6–12 h—*n* (%)	2499 (15.5)	1812 (14.9)	687 (17.5)	
12–24 h—*n* (%)	1088 (6.8)	793 (6.5)	295 (7.5)	
>24 h—*n* (%)	574 (3.6)	418 (3.4)	156 (4)	
Total ischemia time > 12 h—*n* (%)	1662 (10.3)	1211 (10)	451 (11.5)	0.01
Door-to-balloon time, median [25–75th]	400 [25–67]	40 [25–65]	40 [25–70]	0.08
Door-to-balloon time				0.13
<30 min—*n* (%)	6433 (40.0)	4895 (40.2)	1538 (39.2)	
30–60 min—*n* (%)	5259 (32.7)	3997 (32.9)	1262 (32.2)	
>60 min—*n* (%)	4391 (27.3)	3272 (26.9)	1119 (28.6)	
Door-to-balloon time > 30 min—*n* (%)	9650 (60.0)	7269 (59.8)	2381 (60.8)	0.27
***Clinical Presentation***				
Anterior STEMI—*n* (%)	7446 (46.3)	5693 (46.8)	1753 (44.7)	0.03
Out-of-hospital cardiac arrest—*n* (%)	956 (5.9)	746 (6.1)	210 (5.4)	0.08
Cardiogenic shock—*n* (%)	1169 (7.3)	856 (7)	313 (8)	0.05
Rescue PCI for failed thrombolysis—*n* (%)	1099 (6.8)	832 (6.8)	267 (6.8)	0.97
SARS-CoV-2 positivity—*n* (%)	109 (0.7)	80 (0.7)	29 (0.7)	0.58

CAD = Coronary artery disease; STEMI = ST-segment elevation myocardial infarction; PCI = percutaneous coronary intervention; CABG = coronary artery bypass graft.

Time to reperfusion > 12 h (*p* = 0.01), ambulance referral (*p* = 0.03) and cardiogenic shock at presentation (*p* = 0.05) were also more common at presentation in women, but not anterior STEMI (*p* = 0.03). Median door-to-balloon time was, instead, not conditioned by sex.

Anatomic features of the culprit lesion and procedural details are reported in Table 2. As shown, preprocedural TIMI 0 flow (*p* < 0.001), multivessel disease (*p* < 0.001), use of thrombectomy (*p* < 0.001), use of Glycoprotein IIbIIIa inhibitors or cangrelor (*p* < 0.001), radial access (*p* < 0.001) and implantation of DES (*p* = 0.001) were less common among females.

**Figure 1:**
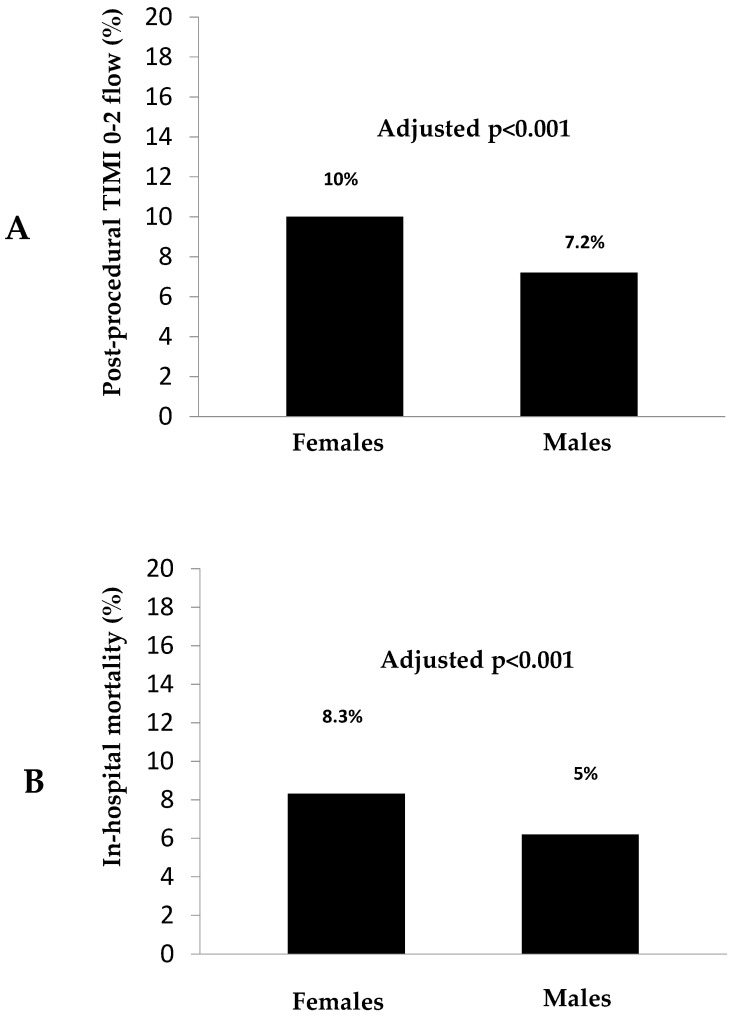
Bar graph showing the impact of sex on postprocedural TIMI 0–2 flow ((**A**), **upper graph**) and in-hospital mortality ((**B**), **lower graph**) in the overall population.

Impaired postprocedural epicardial reperfusion (TIMI 0–2 flow) was observed more frequently in females as compared to males (10% vs. 7.2%; OR [95% CI]= 1.43 [1.26–1.62], *p* < 0.001), as shown in Figure 1A. Results were confirmed at multivariate analysis, after correction for baseline differences, which were entered in the model in-block (adjusted OR [95% CI] = 1.30 [1.13–1.49], *p* < 0.001).

**Table 2:** Angiographic and procedural characteristics.

	Total Population (*n* = 16,083)	Males (*n* = 12,164)	Females (*n* = 3919)	*p*-Value
Radial Access (%)	12,268 (76.3)	9398 (77.3)	2870 (73.2)	<0.001
***Culprit vessel***				<0.001
Left Main—*n* (%)	252 (1.6)	198 (1.6)	54 (1.3)	
Left Anterior Descending Artery—*n* (%)	7358 (45.8)	5608 (46.3)	1750 (44.7)	
Circumflex—*n* (%)	2350 (14.6)	1834 (14.7)	516 (13.2)	
Right Coronary Artery—*n* (%)	6001 (37.3)	4423 (36.4)	1578 (40.3)	
Anterolateral Branch—*n* (%)	41 (0.3)	36 (0.4)	5 (0.1)	
SVG—*n* (%)	79 (0.5)	63 (0.6)	16 (0.4)	
In-stent Thrombosis—*n* (%)	632 (3.9)	501 (4.1)	131 (3.3)	0.03
Multivessel Disease—*n* (%)	7886 (49.0)	6065 (49.9)	1821 (46.5)	<0.001
Preprocedural TIMI 0 flow—*n* (%)	10,731 (66.7)	9543 (78.5)	2971 (75.8)	0.001
Thrombectomy—*n* (%)	2563 (15.9)	2036 (16.7)	527 (13.4)	<0.001
Drug-eluting Stent—*n* (%)	14,254 (88.6)	10,841 (89.1)	3413 (87.1)	0.001
Postprocedural TIMI 3 Flow—*n* (%)	14,821(92.2)	11,292 (92.8)	3529 (90)	<0.001
Gp IIb-IIIa Inhibitors/Cangrelor—*n* (%)	3267 (20.3)	2606 (21.4)	661 (16.9)	<0.001
Mechanical Support—*n* (%)	497 (3.1)	382 (3.1)	115 (2.9)	0.56
***Additional PCI***				<0.001
During the Index Procedure—*n* (%)	1576 (9.8)	1204 (9.9)	372 (9.5)	
Staged—*n* (%)	1696 (10.5)	1331 (10.9)	355 (9.1)	
DAPT Therapy—*n* (%)	15,905 (98.9)	12,031 (98.8)	3874 (98.9)	0.80
In-hospital Death—*n* (%)	938 (5.8)	612 (5)	326 (8.3)	<0.001
30-day Death—*n* (%)	1027 (7.2)	675 (6.2)	352 (10.3)	<0.001

TIMI = Thrombolysis In Myocardial Infarction; DAPT = dual antiplatelet therapy.

As shown in Figure S1, we did not observe a significant age–sex interaction. In fact, poorer postprocedural epicardial reperfusion was observed in females as compared to males across all age categories (unadjusted OR: 1.39, 1.07, 1.16, 1.42, 1.43; *p* int = 0.52).

### In-Hospital and 30-Day Mortality

During hospitalization, 938 (5.8%) patients died, with a significantly higher mortality among women as compared to men (8.3% vs. 5%, OR [95% CI] = 1.32 [1.16–1.5], *p* < 0.001), as depicted in Figure 1B.

A significantly higher in-hospital mortality was observed in 2020 as compared to 2019 (6.5% vs. 5.3%), being higher among females in both years (2020: 10% vs. 5.4%, *p* < 0.001; 2019: 6.9% vs. 4.7%, *p* < 0.001, *p* for interaction = 0.069). The impact of female sex on in-hospital mortality was confirmed via multivariate analysis after correction for baseline confounders (adjusted HR [95% CI] = 1.26 [1.06–1.5], *p* = 0.01).

As shown in Figure S2, we did not observe a significant age–sex interaction. In fact, in-hospital mortality was higher in females across all age categories (unadjusted OR: 1.41, 0.96, 1.24, 1.34, 1.36; *p* int = 0.61).

**Figure 2:**
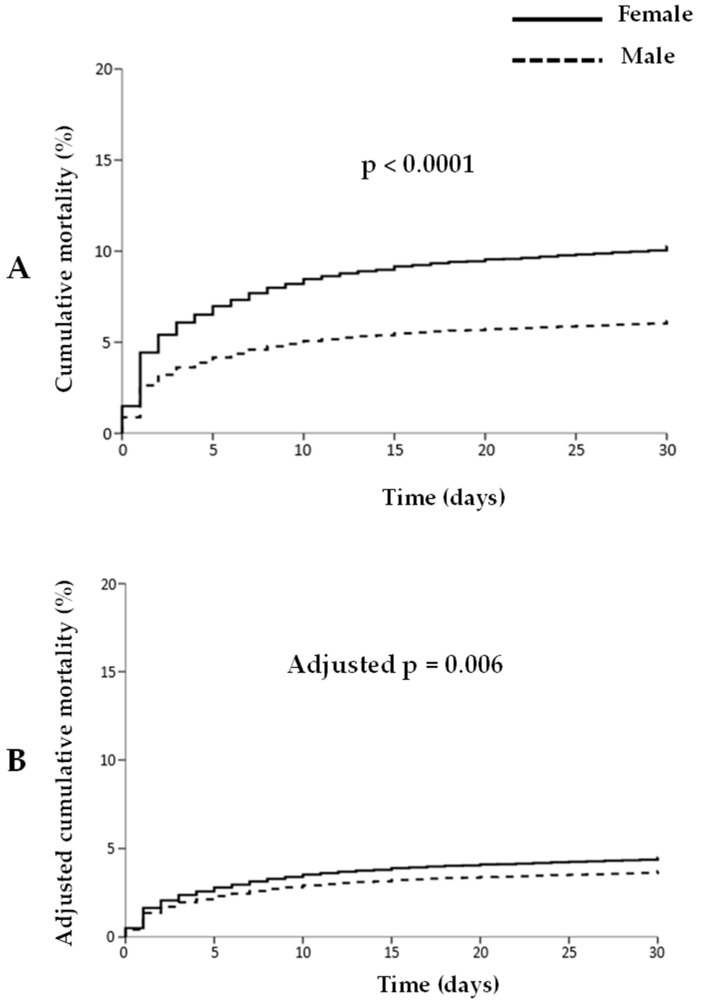
Unadjusted (**A**) and adjusted (**B**) Kaplan–Meier curves showing the risk of mortality at 30 days, according to sex.

Data on 30-day mortality were available in 14,303 patients (88.9%). Female sex was associated with a significantly higher 30-day mortality (10.3% vs. 6.2%, *p* < 0.001; HR [95% CI] = 1.7 [1.5–1.94], *p* < 0.001), as shown in Figure 2. The higher mortality in women was observed both in 2020 (12.1% vs. 6.7%, *p* < 0.001) and 2019 (8.7% vs. 5.8%, *p* < 0.001; *p* for interaction = 0.1) and it was confirmed via multivariate analysis, after correction for baseline confounders (adjusted HR [95% CI] = 1.22 [1.06–1.38], *p* = 0.007). As shown in Figure S3, results were confirmed across all age categories, with no significant age–sex interaction (unadjusted OR: 1.39, 1.0, 1.25, 1.24, 1.39; *p* int = 0.75).

## 4. Discussion

The ISACS-STEMI COVID-19 [22] represents one of the largest STEMI registries worldwide, encompassing more than 16,000 patients undergoing primary PCI with contemporary strategies, thus providing insight into the real-world outcomes of STEMI. The present sub-analysis focused, in particular, on sex-related prognostic discrepancies, which still represent one of the most long-standing unsolved challenges in interventional cardiology [23,24].

Women, in fact, still display poorer prognosis in the context of STEMI, although the independent role of sex in the prediction of mortality is still debated [6,25,26,27,28,29,30,31,32,33,34,35,36,37,38,39,40].

Antoniucci et al. [28], in a population of 1037 patients treated with primary angioplasty, found that female sex was associated with a significantly higher mortality rate, although not confirmed in multivariate analysis. Similar conclusions were achieved by Brodie et al. [29] in a population of 1450 patients treated with primary PCI, as well as by Vacek et al. [26] and De Luca et al. in different study populations, including 1548 [30], 6298 [32], and 1662 [33] STEMI patients, respectively. All these studies supported the concept that the higher mortality among women could be explained by their worse risk profile, including more advanced age and higher rates of comorbidities.

Opposite findings were observed by Vakili et al. [27], who found that, in a population of 1044 patients treated with primary angioplasty, female sex was an independent predictor of mortality. Furthermore, in the large population (more than 50,000 patients) included in the SWEDEHEART registry, Lawesson et al. [35] observed that females displayed higher mortality at short-term follow-up, but not at long-term follow-up.

An analysis from the GRACE registry [41], comparing 7638 women and 19 117 men, showed that women were older than men, had higher rates of cardiovascular risk factors and 6-month mortality. The difference in mortality was no longer statistically significant after adjustment for age and the extent of disease. However, these analyses were conducted on a population treated between 20 and 30 years ago, and across the entire spectrum of acute coronary syndrome types.

Therefore, caution should be applied before translating these findings to the actual STEMI population, as most large-scale studies were limited by the use of bare metal stents or first-generation DES, suboptimal antithrombotic therapies and prevalence of femoral approach, all factors that have been demonstrated to negatively impact outcomes and that have been addressed in current practice [42,43,44,45,46,47,48].

In fact, the large retrospective New South Wales cohort, encompassing 29,435 patients with STEMI (of whom 28.8% were females) from 2011 to 2020, showed a rapid increase in the percentage of women undergoing timely PCI and a subsequent larger reduction in mortality and major cardiovascular events (MACEs) in females (0.8 percentage points by year in females vs. 0.5 in males for cardiovascular death). Nevertheless, the risk of death or adverse events at 12-month follow-up remained higher among women [49]. Moreover, in a subgroup analysis of the HORIZONS-AMI trial [50], which randomized 3602 patients (23.4% women) with STEMI to receive bivalirudin or heparin plus glycoprotein IIb/IIIa inhibitors and percutaneous coronary intervention (PCI) with drug-eluting or bare metal stents, female sex was not an independent predictor of long-term MACE at 3 years after adjusting for differences in baseline and treatment characteristics, while it remained an independent predictor of major bleeding at 3 years.

Similarly, the recent study by Paradossi et al. [38] and two large-scale meta-analyses, including 891,585 [51] and 358,140 [52] patients, respectively, concluded that female sex was independently associated with higher in-hospital and long-term mortality, driven by comorbidities, especially diabetes mellitus, longer treatment delays and suboptimal care.

In the present analysis of the ISACS-STEMI COVID-19 registry [22], we observed, in accordance with all previous studies, that females had a worse baseline risk profile, with more advanced age, higher rates of diabetes, hypertension, hypercholesterolemia, but were less often smokers and less often had a previous cardiovascular event. Women had a longer ischemia time, derived from a longer pre-hospital delay, since no difference was observed in terms of door-to-balloon time, and presented more often with cardiogenic shock, despite a lower prevalence of anterior STEMI. Radial access, thrombectomy and Gp IIb-IIIa inhibitors were less frequently used for females, in accordance with the pooled data of Cheo et al. [51]. Delays in revascularization and less aggressive antithrombotic therapies translated into more frequent suboptimal epicardial reperfusion among women, although multivessel coronary disease and impaired preprocedural recanalization were more often observed among men. Biological differences in the atherothrombotic process and microcirculation may have potentially accounted for such differences in outcomes. These results were in line with a previous study by De Luca et al. [33], where female sex was associated with impaired reperfusion and more frequent distal embolization.

Impaired postprocedural epicardial reperfusion, however, can represent only one of the several potential explanations for the higher in-hospital and 30-day mortality observed in the current analysis among females vs. males, which was confirmed after correction for all baseline confounding factors.

Invasive studies with the use of intravascular imaging (OCT) have recently demonstrated a significantly higher prevalence of high-risk plaques among women as compared to men [13], which may be a contributing factor in explaining the observed angiographic findings in terms of reperfusion and distal embolization described in our current and previous populations of women.

In previous reports, which are in line with our study, female sex was associated with more advanced Killip class at presentation and larger occurrence of cardiogenic shock, despite the lower prevalence of previous infarction, anterior infarction location, and similar left ventricular function [6,53,54]. Worse diastolic function in women in comparison with men, caused by their more advanced age at presentation, could be a plausible reason to account for these results [55]. The impaired outcome in women may also be explained by the smaller coronary size and postprocedural minimal lumen diameter, as reported in previous studies, since we could not assess vessel diameter in our study [33]. Moreover, more delayed presentation and atypical symptoms have been reported more frequently among women, often delaying diagnosis and management.

Our results were also confirmed across different ages: we found that female sex was associated with worse outcomes and impaired postprocedural reperfusion, which was observed at a similar magnitude irrespective of age. Indeed, previous reports investigating this issue reached contrasting results, with some authors showing a significant age–sex interaction in terms of mortality [56,57], which was not confirmed by other studies [58].

In addition, as the present study was performed during the COVID-19 pandemic, additional considerations can be made regarding its direct and indirect effects on survival. In fact, the nearly 16% reduction in patients admitted for STEMI and primary PCI procedures, observed during the pandemic, was not conditional on gender [19]. Nevertheless, this did not translate into any prognostic benefit for women. Furthermore, while we observed a larger difference in absolute mortality in 2020 as compared to 2019, the interaction was not statistically significant.

Female patients with STEMI are generally older and frail, with increased rates of comorbidities that can enhance both thrombotic and bleeding risk [6,24,25,26,27,28,29,30,31,32,33,34,35,36,37,38,39,40,59]. Although we could not assess hemorrhagic complications using our registry, bleeding can also be claimed to increase mortality among women with STEMI. In two previous large-scale trials [6,60], the higher mortality in women after interventional treatment for STEMI was explained by the differences in body size and clinical risk factors, increasing both access site and non-access site bleeding. In effect, the implementation of the most updated guidelines, with a preferential use of the transradial approach, has significantly contributed to overcoming these complications, although its routine use for interventional procedures is generally more challenging, especially among elderly women, presenting with smaller and more tortuous radial arteries [61].

The importance of these studies is to reaffirm the biological, sex-specific differences in the pathogenesis of cardiovascular disease and to consider the persisting prognostic gap between women and men presenting with STEMI, despite the use of primary PCI and treatment with the best standard of care in terms of revascularization and antithrombotic therapies. In fact, future large-scale trials dedicated to women are certainly required, allowing for a refinement of interventional and pharmacological strategies, tailored to account for the higher complexity of female patients.

## 5. Limitations

The first limitation relates to the study design, which did not involve prospective data collection or follow-up. Therefore, we could not assess any causal relationship between gender and outcomes. Nevertheless, the retrospective analysis was considered advisable due to the pandemic constraints and in order to capture the most comprehensive cohort of unselected real-life STEMI patients, offering an overview of contemporary clinical practice. Moreover, we believe that the potential limitations due to missing or irretrievable data were largely compensated for by the size of the population included and by the high rate of complete cases (>95%). In addition, despite the discrepancy in the proportion of males and females, the large sample size warranted sufficient statistical power for the study analysis, including multivariate models. However, our female population presented with a higher-risk profile (older age, more comorbidities, longer ischemic time, and more cardiogenic shock) and residual confounding remained a significant concern despite multivariable adjustment. Indeed, by including only patients admitted for primary angioplasty, we could not evaluate the rates of pre-hospital death, which could have differed according to sex, although we did not find any difference in out-of-hospital cardiac arrest. In addition, we did not include the more complex patients who were deemed at too high a risk to undergo an invasive approach, potentially introducing an unmeasurable selection bias.

In addition, we did not collect data on bleeding and access-site complications, which have been identified as major determinants of the worst outcomes in female patients. Certain laboratory parameters, including the rate of LDL cholesterol, were not available in the majority of the patients; therefore, we decided not to include them in the analysis. However, we considered that metabolic parameters and thepharmacological therapy at discharge could have provided a more significant impact on long-term prognosis, rather than at 30 days. Similarly, risk stratification tools, such as HAS-BLED or PRECISE-DAPT scores, were not calculated at the time of recruitment in all the patients, and therefore, we cannot provide more data about the estimated ischemic and bleeding risk of our patients.

Finally, we could not provide data on the angiographic features of the culprit lesion, since calcification, tortuosity and smaller reference diameter generally occur more frequently among women in consequence ofspontaneous coronary dissections or MINOCA [62], which could have conditioned the lower rate of stenting observed in our study and, therefore, the final prognosis.

**Conclusions.** Among STEMI patients being treated with the most updated standard of care for primary percutaneous coronary intervention, female sex is still associated with higher complexity and impaired prognosis, displaying suboptimal epicardial reperfusion and increased in-hospital and 30-day mortality.

## Data Availability

The raw data supporting the conclusions of this article will be made available by the authors on request.

## Supplementary Materials

The following supporting information can be downloaded at: https://www.mdpi.com/article/10.3390/jcm15103560/s1, Figure S1:Gender and postprocedural TIMI 0-2 flow interaction across age categories.; Figure S2: Gender and in-hospital mortality interaction across age categories; Figure S3: Gender and 30-day mortality interaction across age categories.

## Abbreviations

PCI	Percutaneous coronary intervention
STEMI	ST-segment elevation myocardial infarction
DTB	Door-to-balloon time
ACS	Acute coronary syndrome
DES	Drug-eluting stent
GPIIbIIIa	Glycoprotein IIbIIIa inhibitors

## References

[1] De Luca G., Damen S.A., Camaro C., Benit E., Verdoia M., Rasoul S., Liew H.B., Polad J., Ahmad W.A., Zambahari R. (2019). Final results of the randomised evaluation of short-term dual antiplatelet therapy in patients with acute coronary syndrome treated with a new-generation stent (REDUCE trial). EuroIntervention.

[2] Chichareon P., Modolo R., Collet C., Tenekecioglu E., Vink M.A., Oh P.C., Ahn J.M., Musto C., Díaz de la Llera L.S., Cho Y.S. (2019). Efficacy and Safety of Stents in ST-Segment Elevation Myocardial Infarction. J. Am. Coll. Cardiol..

[3] De Luca G., Smits P., Hofma S.H., Di Lorenzo E., Vlachojannis G.J., Van’t Hof A.W.J., van Boven A.J., Kedhi E., Stone G.W., Suryapranata H. (2017). Drug-Eluting Stent in Primary Angioplasty (DESERT 3) cooperation. Everolimus eluting stent vs first generation drug-eluting stent in primary angioplasty: A pooled patient-level meta-analysis of randomized trials. Int. J. Cardiol..

[4] Niehe S.R., Vos N.S., Van Der Schaaf R.J., Amoroso G., Herrman J.R., Patterson M.S., Slagboom T., Vink M.A. (2022). Two-Year Clinical Outcomes of the REVELATION Study: Sustained Safety and Feasibility of Paclitaxel-Coated Balloon Angioplasty Versus Drug-Eluting Stent in Acute Myocardial Infarction. J. Invasive Cardiol..

[5] Lansky A.J., Tirziu D., Moses J.W., Pietras C., Ohman E.M., O’Neill W.W., Ekono M.M., Grines C.L., Parise H. (2022). Impella Versus Intra-Aortic Balloon Pump for High-Risk PCI: A Propensity-Adjusted Large-Scale Claims Dataset Analysis. Am. J. Cardiol..

[6] Lansky A.J., Pietras C., Costa R.A., Tsuchiya Y., Brodie B.R., Cox D.A., Aymong E.D., Stuckey T.D., Garcia E., Tcheng J.E. (2005). Gender differences in outcomes after primary angioplasty versus primary stenting with and without abciximab for acute myocardial infarction: Results of the Controlled Abciximab and Device Investigation to Lower Late Angioplasty Complications (CADILLAC) trial. Circulation.

[7] Riehle L., Gothe R.M., Ebbinghaus J., Maier B., Bruch L., Röhnisch J.U., Schühlen H., Fried A., Stockburger M., Theres H. (2023). Implementation of the ESC STEMI guidelines in female and elderly patients over a 20-year period in a large German registry. Clin. Res. Cardiol..

[8] Negro F., Verdoia M., Tonon F., Nardin M., Kedhi E., De Luca G. (2020). Novara Atherosclerosis Study Group (NAS). Impact of gender on immature platelet count and its relationship with coronary artery disease. J. Thromb. Thrombolysis.

[9] Barbieri L., Verdoia M., Schaffer A., Marino P., Suryapranata H., De Luca G. (2015). Novara Atherosclerosis Study Group (NAS). Impact of sex on uric acid levels and its relationship with the extent of coronary artery disease: A single-centre study. Atherosclerosis.

[10] Verdoia M., Schaffer A., Barbieri L., Di Giovine G., Marino P., Suryapranata H., De Luca G. (2015). Novara Atherosclerosis Study Group (NAS). Impact of gender difference on vitamin D status and its relationship with the extent of coronary artery disease. Nutr. Metab. Cardiovasc. Dis..

[11] Jia K., Luo X., Yi J., Zhang C. (2024). Hormonal influence: Unraveling the impact of sex hormones on vascular smooth muscle cells. Biol. Res..

[12] Verdoia M., Suryapranata H., Damen S., Camaro C., Benit E., Barbieri L., Rasoul S., Liew H.B., Polad J., Ahmad W.A.W. (2021). Gender differences with short-term vs 12 months dual antiplatelet therapy in patients with acute coronary syndrome treated with the COMBO dual therapy stent: 2-years follow-up results of the REDUCE trial. J. Thromb. Thrombolysis.

[13] Volleberg R.H.J.A., Mol J.Q., Belkacemi A., Hermanides R.S., Meuwissen M., Protopopov A.V., Laanmets P., Krestyaninov O.V., Dennert R., Oemrawsingh R.M. (2024). Sex differences in plaque characteristics of fractional flow reserve-negative non-culprit lesions after myocardial infarction. Atherosclerosis.

[14] Tousoulis D., Guzik T., Padro T., Duncker D.J., De Luca G., Eringa E., Vavlukis M., Antonopoulos A.S., Katsimichas T., Cenko E. (2022). Mechanisms, therapeutic implications, and methodological challenges of gut microbiota and cardiovascular diseases: A position paper by the ESC Working Group on Coronary Pathophysiology and Microcirculation. Cardiovasc. Res..

[15] Verdoia M., Pergolini P., Rolla R., Nardin M., Schaffer A., Barbieri L., Marino P., Bellomo G., Suryapranata H., De Luca G. (2016). Advanced age and high-residual platelet reactivity in patients receiving dual antiplatelet therapy with clopidogrel or ticagrelor. J. Thromb. Haemost..

[16] Ortega R.F., Mehran R., Morice M.C. (2020). The Opportunity of Women as One. JACC Case Rep..

[17] King A. (2011). The heart of a woman: Addressing the gender gap in cardiovascular disease. Nat. Rev. Cardiol..

[18] Stramba-Badiale M., Fox K.M., Priori S.G., Collins P., Daly C., Graham I., Jonsson B., Schenck-Gustafsson K., Tendera M. (2006). Cardiovascular diseases in women: A statement from the policy conference of the European Society of Cardiology. Eur. Heart J..

[19] De Luca G., Manzo-Silberman S., Algowhary M., Uguz B., Oliveira D.C., Ganyukov V., Busljetik O., Cercek M., Okkels L., Loh P.H. (2023). Gender Difference in the Effects of COVID-19 Pandemic on Mechanical Reperfusion and 30-Day Mortality for STEMI: Results of the ISACS-STEMI COVID-19 Registry. J. Clin. Med..

[20] Doolub G., Tonino P.A.L., Kedev S., Monségu J., Paradies V., Austin D., Spanó F., Roffi M., Fröbert O., von Birgelen C. (2023). Impact of Sex on Clinical Outcomes in Patients undergoing Complex Percutaneous Coronary Angioplasty (from the e-ULTIMASTER Study). Am. J. Cardiol..

[21] Park H.W., Han S., Park G.M., Ann S.H., Suh J., Kim Y.G., Lee S.W., Kim Y.H. (2020). Sex-related impacts on clinical outcomes after percutaneous coronary intervention. Sci. Rep..

[22] De Luca G., Verdoia M., Cercek M., Jensen L.O., Vavlukis M., Calmac L., Johnson T., Ferrer G.R., Ganyukov V., Wojakowski W. (2020). Impact of COVID-19 Pandemic on Mechanical Reperfusion for Patients with STEMI. J. Am. Coll. Cardiol..

[23] De Luca G., Cercek M., Jensen L.O., Vavlukis M., Calmac L., Johnson T., Roura IFerrer G., Ganyukov V., Wojakowski W., von Birgelen C. (2020). Impact of COVID-19 pandemic and diabetes on mechanical reperfusion in patients with STEMI: Insights from the ISACS STEMI COVID 19 Registry. Cardiovasc. Diabetol..

[24] De Luca G., Algowhary M., Uguz B., Oliveira D.C., Ganyukov V., Zimbakov Z., Cercek M., Jensen L.O., Loh P.H., Calmac L. (2022). COVID-19 pandemic, mechanical reperfusion and 30-day mortality in ST elevation myocardial infarction. Heart.

[25] Dawson L.P., Nehme E., Nehme Z., Davis E., Bloom J., Cox S., Nelson A.J., Okyere D., Anderson D., Stephenson M. (2023). Sex Differences in Epidemiology, Care, and Outcomes in Patients with Acute Chest Pain. J. Am. Coll. Cardiol..

[26] Vacek J.L., Rosamond T.L., Kramer P.H., Crouse L.J., Porter C.B., Robuck O.W., White J.L., Beauchamp G.D. (1993). Sex-related differences in patients undergoing direct angioplasty for acute myocardial infarction. Am. Heart J..

[27] Vakili B.A., Kaplan R.C., Brown D.L. (2001). Sex-based differences in early mortality of patients undergoing primary angioplasty for first acute myocardial infarction. Circulation.

[28] Antoniucci D., Valenti R., Moschi G., Migliorini A., Trapani M., Santoro G.M., Bolognese L., Dovellini E.V. (2001). Sex-based differences in clinical and angiographic outcomes after primary angioplasty or stenting for acute myocardial infarction. Am. J. Cardiol..

[29] Brodie B.R. (1999). Why is mortality rate after percutaneous transluminal coronary angioplasty higher in women?. Am. Heart J..

[30] De Luca G., Suryapranata H., Dambrink J.H., Ottervanger J.P., van ‘t Hof A.W., Zijlstra F., Hoorntje J.C., Gosselink A.T., de Boer M.J. (2004). Sex-related differences in outcome after ST-segment elevation myocardial infarction treated by primary angioplasty: Data from the Zwolle Myocardial Infarction study. Am. Heart J..

[31] Goldberg R.J., Gorak E.J., Yarzebski J., Hosmer D.W., Dalen P., Gore J.M., Alpert J.S., Dalen J.E. (1993). A communitywide perspective of sex differences and temporal trends in the incidence and survival rates after acute myocardial infarction and out-of-hospital deaths caused by coronary heart disease. Circulation.

[32] De Luca G., Verdoia M., Dirksen M.T., Spaulding C., Kelbæk H., Schalij M., Thuesen L., Hoeven Bv Vink M.A., Kaiser C., Musto C. (2013). Gender-related differences in outcome after BMS or DES implantation in patients with ST-segment elevation myocardial infarction treated by primary angioplasty: Insights from the DESERT cooperation. Atherosclerosis.

[33] De Luca G., Gibson C.M., Gyöngyösi M., Zeymer U., Dudek D., Arntz H.R., Bellandi F., Maioli M., Noc M., Zorman S. (2010). Gender-related differences in outcome after ST-segment elevation myocardial infarction treated by primary angioplasty and glycoprotein IIb-IIIa inhibitors: Insights from the EGYPT cooperation. J. Thromb. Thrombolysis.

[34] De Rosa R., Morici N., De Luca G., De Luca L., Ferri L.A., Piatti L., Tortorella G., Grosseto D., Franco N., Misuraca L. (2021). Association of Sex with Outcome in Elderly Patients with Acute Coronary Syndrome Undergoing Percutaneous Coronary Intervention. Am. J. Med..

[35] Lawesson S.S., Alfredsson J., Fredrikson M., Swahn E. (2013). A gender perspective on short- and long term mortality in ST-elevation myocardial infarction--a report from the SWEDEHEART register. Int. J. Cardiol..

[36] Ng V.G., Mori K., Costa R.A., Kish M., Mehran R., Urata H., Saku K., Stone G.W., Lansky A.J. (2016). Impact of gender on infarct size, ST-segment resolution, myocardial blush and clinical outcomes after primary stenting for acute myocardial infarction: Substudy from the EMERALD trial. Int. J. Cardiol..

[37] Lansky A., Baron S.J., Grines C.L., Tremmel J.A., Al-Lamee R., Angiolillo D.J., Chieffo A., Croce K., Jacobs A.K., Madan M. (2022). SCAI Expert Consensus Statement on Sex-Specific Considerations in Myocardial Revascularization. J. Soc. Cardiovasc. Angiogr. Interv..

[38] Paradossi U., Taglieri N., Massarelli G., Palmieri C., De Caterina A.R., Bruno A.G., Taddei A., Nardi E., Ghetti G., Palmerini T. (2022). Female gender and mortality in ST-segment-elevation myocardial infarction treated with primary PCI. J. Cardiovasc. Med..

[39] De Luca G., Parodi G., Sciagrà R., Bellandi B., Verdoia M., Vergara R., Migliorini A., Valenti R., Antoniucci D. (2013). Relation of gender to infarct size in patients with ST-segment elevation myocardial infarction undergoing primary angioplasty. Am. J. Cardiol..

[40] Bucholz E.M., Butala N.M., Rathore S.S., Dreyer R.P., Lansky A.J., Krumholz H.M. (2014). Sex differences in long-term mortality after myocardial infarction: A systematic review. Circulation.

[41] Dey S., Flather M.D., Devlin G., Brieger D., Gurfinkel E.P., Steg P.G., Fitzgerald G., Jackson E.A., Eagle K.A. (2009). Global Registry of Acute Coronary Events investigators. Sex-related differences in the presentation, treatment and outcomes among patients with acute coronary syndromes: The Global Registry of Acute Coronary Events. Heart.

[42] Costa F., Montalto C., Branca M., Hong S.J., Watanabe H., Franzone A., Vranckx P., Hahn J.Y., Gwon H.C., Feres F. (2023). Dual antiplatelet therapy duration after percutaneous coronary intervention in high bleeding risk: A meta-analysis of randomized trials. Eur. Heart J..

[43] De Luca G., Bellandi F., Huber K., Noc M., Petronio A.S., Arntz H.R., Maioli M., Gabriel H.M., Zorman S., DECarlo M. (2011). Early glycoprotein IIb-IIIa inhibitors in primary angioplasty-abciximab long-term results (EGYPT-ALT) cooperation: Individual patient’s data meta-analysis. J. Thromb. Haemost..

[44] Verdoia M., Schaffer A., Barbieri L., Cassetti E., Piccolo R., Galasso G., Marino P., Sinigaglia F., De Luca G. (2014). Benefits from new ADP antagonists as compared with clopidogrel in patients with stable angina or acute coronary syndrome undergoing invasive management: A meta-analysis of randomized trials. J. Cardiovasc. Pharmacol..

[45] De Luca G., Schaffer A., Wirianta J., Suryapranata H. (2013). Comprehensive meta-analysis of radial vs femoral approach in primary angioplasty for STEMI. Int. J. Cardiol..

[46] De Luca G., Navarese E.P., Suryapranata H. (2013). A meta-analytic overview of thrombectomy during primary angioplasty. Int. J. Cardiol..

[47] De Luca G., Navarese E.P., Cassetti E., Verdoia M., Suryapranata H. (2011). Meta-analysis of randomized trials of glycoprotein IIb/IIIa inhibitors in high-risk acute coronary syndromes patients undergoing invasive strategy. Am. J. Cardiol..

[48] Schoos M.M., De Luca G., Dangas G.D., Clemmensen P., Ayele G.M., Mehran R., Stone G.W. (2016). Impact of time to treatment on the effects of bivalirudin vs. glycoprotein IIb/IIIa inhibitors and heparin in patients undergoing primary percutaneous coronary intervention: Insights from the HORIZONS-AMI trial. EuroIntervention.

[49] Kazi S., Marschner S., Min H., Quintans D., Chong J.J., Khan E., Brieger D.B., Chow C.K. (2025). Sex differences in management and outcomes of people with ST-elevation myocardial infarction, New South Wales, 2011–2020: A retrospective cohort study. Med. J. Aust..

[50] Yu J., Mehran R., Grinfeld L., Xu K., Nikolsky E., Brodie B.R., Witzenbichler B., Kornowski R., Dangas G.D., Lansky A.J. (2015). Sex-based differences in bleeding and long term adverse events after percutaneous coronary intervention for acute myocardial infarction: Three year results from the HORIZONS-AMI trial. Catheter. Cardiovasc. Interv..

[51] Cheo H.-M., Lee F., Lee A., Ong X., Koh L.-P., Chan M.Y., Wee I., Ong J., Sia C.-H. (2026). A Global Systematic Review and Meta-Analysis of Sex Disparities in Outcomes in Patients with ST-segment Elevation Myocardial Infarction. medRxiv.

[52] Di Pietro G., Improta R., De Filippo O., Birtolo L.I., Bruno E., Sardella G., Vizza C.D., D’Ascenzo F., Stefanini G., Mancone M. (2025). Long-term clinical impact of sex disparities in patients with ST elevation acute myocardial infarction: A systematic review and meta-analysis of adjusted observational studies. BMJ Open..

[53] Woodfield S.L., Lundergan C.F., Reiner J.S., Thompson M.A., Rohrbeck S.C., Deychak Y., Smith J.O., Burton J.R., McCarthy W.F., Califf R.M. (1997). Gender and acute myocardial infarction: Is there a different response to thrombolysis?. J. Am. Coll. Cardiol..

[54] Zoni C.R., D’Imperio H., Zapata G., Charask A., Macín S.M., Castillo Costa Y., Ravi Y., Gagliardi J., Perna E.R. (2024). Researchers of the ARGEN-IAM-ST Registry. Heart Failure at Admission Complicating ST-Elevation Myocardial Infarction in a Middle-Income Country. Experience of the ARGEN-IAM-ST Registry. Curr. Probl. Cardiol..

[55] Fischer M., Baessler A., Hense H.W., Hengstenberg C., Muscholl M., Holmer S., Döring A., Broeckel U., Riegger G., Schunkert H. (2003). Prevalence of left ventricular diastolic dysfunction in the community: Results from a Doppler echocardiographic-based survey of a population sample. Eur. Heart J..

[56] Bangalore S., Fonarow G.C., Peterson E.D., Hellkamp A.S., Hernandez A.F., Laskey W., Peacock W.F., Cannon C.P., Schwamm L.H., Bhatt D.L. (2012). Age and gender differences in quality of care and outcomes for patients with ST-segment elevation myocardial infarction. Am. J. Med..

[57] Zheng X., Dreyer R.P., Hu S., Spatz E.S., Masoudi F.A., Spertus J.A., Nasir K., Li X., Li J., Wang S. (2015). Age-specific gender differences in early mortality following ST-segment elevation myocardial infarction in China. Heart.

[58] De Luca L., Marini M., Gonzini L., Boccanelli A., Casella G., Chiarella F., De Servi S., Di Chiara A., Di Pasquale G., Olivari Z. (2016). Contemporary Trends and Age-Specific Sex Differences in Management and Outcome for Patients with ST-Segment Elevation Myocardial Infarction. J. Am. Heart Assoc..

[59] Timmer J.R., van der Horst I.C., De Luca G., Ottervanger J.P., Hoorntje J.C., de Boer M.J., Suryapranata H., Dambrink J.H., Gosselink M., Zijlstra F. (2005). Comparison of myocardial perfusion after successful primary percutaneous coronary intervention in patients with ST-elevation myocardial infarction with versus without diabetes mellitus. Am. J. Cardiol..

[60] Daly C., Clemens F., Lopez Sendon J.L., Tavazzi L., Boersma E., Danchin N., Delahaye F., Gitt A., Julian D., Mulcahy D. (2006). Gender differences in the management and clinical outcome of stable angina. Circulation.

[61] Sattar Y., Song D., Kompella R., Arshad J., Zghouzi M., Mir T., Ullah W., Elgendy I.Y., Alraies M.C. (2022). Meta-Analysis Comparing Gender-Based Cardiovascular Outcomes of Transradial Versus Transfemoral Access of Percutaneous Coronary Intervention. Am. J. Cardiol..

[62] Ciliberti G., Verdoia M., Merlo M., Zilio F., Vatrano M., Bianco F., Mancone M., Zaffalon D., Bonci A., Boscutti A. (2021). Pharmacological therapy for the prevention of cardiovascular events in patients with myocardial infarction with non-obstructed coronary arteries (MINOCA): Insights from a multicentre national registry. Int. J. Cardiol..

