# Supplementary material for: Persisting Sex Discrepancies in Short-Term Outcomes of Patients with ST-Segment Myocardial Infarction: Results of the ISACS-STEMI COVID-19 Registry
Source: J Clin Med. 2026 May 7;15(10):3560. doi: 10.3390/jcm15103560 (PMC13207220; doi:10.3390/jcm15103560)
Supplement: Supplementary file 1 [file jcm-15-03560-s001.zip › jcm-4208229-supplementary.pdf]

Supplementary Materials

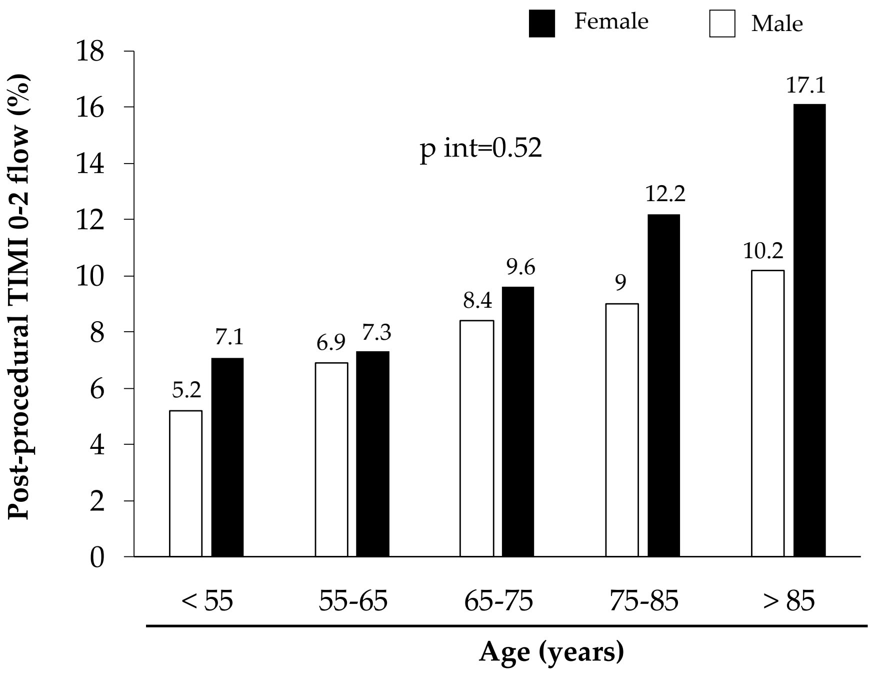

Figure S1. Bar graphs showing the absence of interaction between female gender and postprocedural TIMI 0-2 flow across age categories.

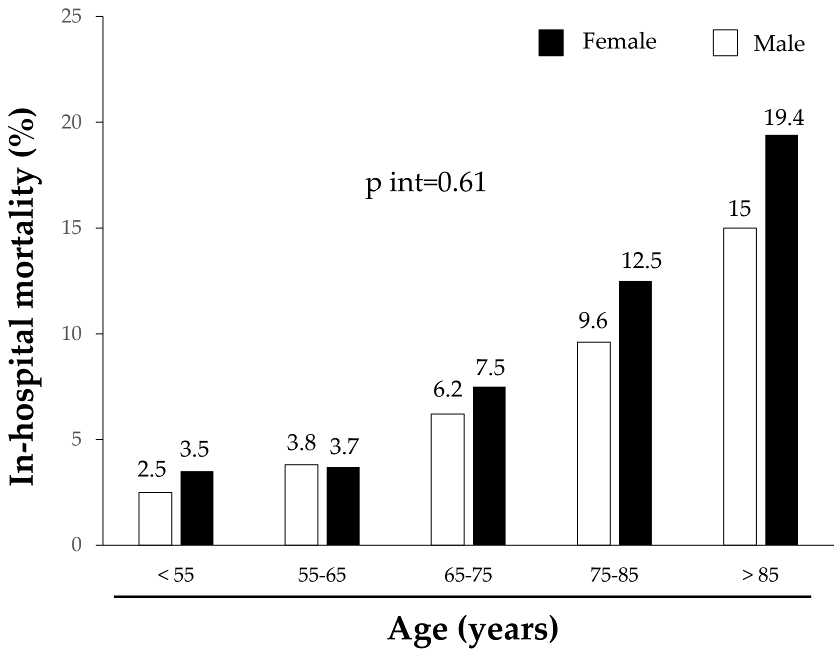

Figure S2. Bar graphs showing the absence of interaction between female gender and in-hospital mortality across age categories.

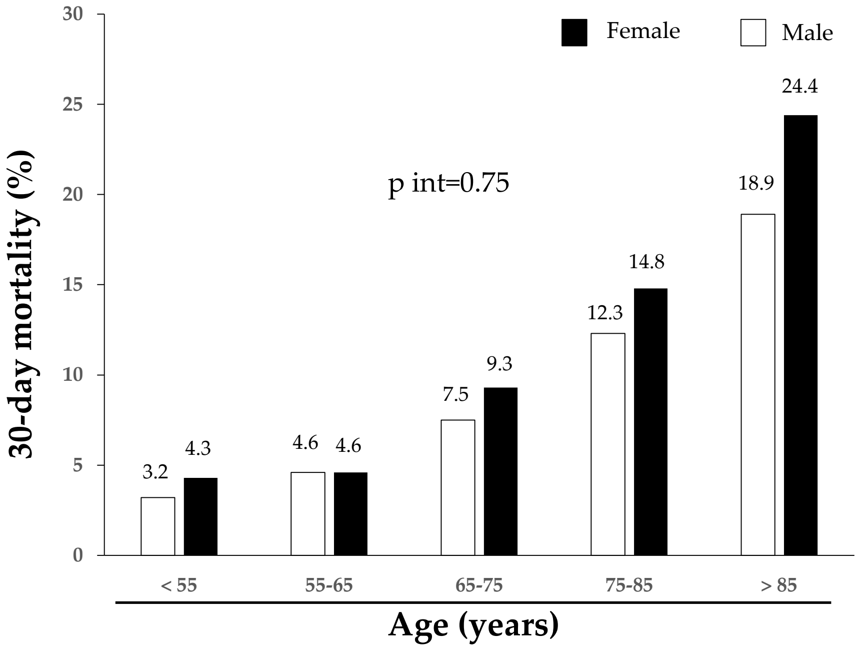

Figure S3. Bar graphs showing the absence of interaction between female gender and 30-day mortality across age categories.
